# Toxin Detection by Surface Plasmon Resonance

**DOI:** 10.3390/s9031339

**Published:** 2009-02-26

**Authors:** Vesna Hodnik, Gregor Anderluh

**Affiliations:** University of Ljubljana, Biotechnical Faculty, Department of Biology, Večna pot 111, 1000 Ljubljana, Slovenia; E-Mail: vesna.hodnik@bf.uni-lj.si (V.H.)

**Keywords:** Surface plasmon resonance, toxins, detection, mycotoxins, ricin, enterotoxin, aflatoxin, atrazine

## Abstract

Significant efforts have been invested in the past years for the development of analytical methods for fast toxin detection in food and water. Immunochemical methods like ELISA, spectroscopy and chromatography are the most used in toxin detection. Different methods have been linked, e.g. liquid chromatography and mass spectrometry (LC-MS), in order to detect as low concentrations as possible. Surface plasmon resonance (SPR) is one of the new biophysical methods which enables rapid toxin detection. Moreover, this method was already included in portable sensors for on-site determinations. In this paper we describe some of the most common methods for toxin detection, with an emphasis on SPR.

## 1. Introduction

The great diversity of toxins represents a big challenge when effective detection is needed. Different chemical and physicochemical characteristics of the analytes lead to specific extraction, sample cleanup and detection. Detection methods are, therefore, specific for individual toxins or groups of similar toxins. Fast and accurate methods are needed in order to deal with the increasing number of toxins of interest. The analyses should avoid large costs and be simple to perform. Faster acquisition of results can be accomplished with portable biosensors, which enable rapid detection outside laboratories.

## 2. Methods for Toxin Detection

Enzyme-linked immunosorbent assays (ELISA) are commonly performed for fast screening of the samples. The advantage of immunoassays is that detection is finished in few hours and no special sample preparation is needed. ELISA has great selectivity and sensitivity, is easy to perform and offers the option of simultaneous detection of numerous samples. Many immunoassays for different toxic molecules have been developed [1,2]. Immunoassays can be linked with other methods like in botulinum toxin detection. Phillips and Abbott recently reported the use of an antibody-based assay similar to an ELISA but utilizing electrochemiluminiscent technology as an alternative to the mouse bioassay for testing food samples [3]. Micheli *et al.* constructed disposable electrochemical aflatoxin M1 immunosensors, which can combine the high selectivity of immunoanalysis with the ease of the electrochemical probes. The electrochemical immunosensors were fabricated by immobilising the antibodies directly on the surface of screen-printed electrodes, and allowing the competition to occur between free aflatoxin M1 and that conjugated with horseradish peroxidase (HRP) enzyme. The electrochemical technique chosen was chronoamperometry. A better detection limit and shorter analysis time were achieved in comparison to the classical spectrophotometric procedure [4].

Another immunosensor was developed for the detection of nitroaromatics and the pesticides diuron and atrazine. An analyte-specific antibody was immobilized on a gold surface of pyramidal structure inside an exchangeable single-use chip, which hosts also the enzyme-tracer and the sample reservoirs. The competition between the enzyme-tracer and the analyte for the antigen-binding sites of the antibodies finally yields a chemiluminescence signal that is inversely proportional to the concentration of analyte in the given range of detection [5].

The mouse bioassay used to be one of the most used methods for toxin determination. In this test the toxin or sample contaminated by the toxin is injected into mice, which are monitored for any physiological changes. This test is expensive, time consuming, the results may be variable and ethically controversial. In last decades significant effort were, therefore, invested in the development of new *in vitro* biophysical methods.

### 2.1. Physical and Biophysical Methods

Spectroscopic methods exploit different optical characteristics of molecules in different range of electromagnetic spectrum. The presence of Cyanobacteria toxic metabolites in natural water was determined by exploiting the color change of bromine thymol blue indicator [6]. Ho *et al.* used fluorescence for the detection of cholera toxin [7].

Mycotoxins are secondary metabolites produced by filamentous fungi that can have toxic effects on vertebrates. In order to improve food safety the presence of mycotoxins must be determined during the process of food and feed preparation. The fast screening is most conventionally done with ELISA. Quantitative methods of analysis for most mycotoxins use immunoaffinity clean-up with high performance liquid chromatography (HPLC) separation in combination with UV and/or fluorescence detection. There is a strong trend towards the use of multi-mycotoxin methods for the simultaneous analysis of several of the important mycotoxins, which is best achieved by LC–MS/MS (liquid chromatography with tandem mass spectrometry) [8].

Another physical method that can be used in toxin detection is amperometry. The principle of amperometry is based on the measurement of the current between the two electrodes, which is induced by a redox reaction at one of the electrode. The conditions are chosen in such a way that the current is directly proportional to the concentration of a redox active species in the analyte solution. An amperometric sensor system for measuring okadaic acid, a diarrheic shellfish poisoning toxin, has been developed recently [9].

Quartz crystal microbalance sensors for the detection of ricin were presented by Stine *et al.* [10]. Antibodies have been used traditionally as the detection molecules for these types of biosensors. However, recent studies have shown that glycosphingolipids may be used for recognition of certain protein toxins, including ricin. Glycosphingolipids had lower detection limits (5 μg/mL), approximately five times lower than were found for antibodies (25 μg/mL) [10]. Quartz crystal microbalance and amperometry detection were used in many cases for the detection of low molecular weight toxins in environment and food [11–13].

## 3. Surface Plasmon Resonance

### 3.1. Detection Principle

One of the methods that offer quick toxin detection is surface plasmon resonance (SPR). This is a relatively new technique, which became more popular with the commercialization of biosensors by the company Biacore in the 90’s. SPR is used in biochemistry to study molecular interactions and it provides significant advantages over other biophysical approaches [14–16]. SPR allows the kinetic parameters of the molecular interaction to be determined, but it is also used for quantitative purposes, i.e. for the determination of unknown concentrations of analytes in complex samples. The significant advantage over other biophysical approaches is that the interaction is studied in real time and without the need of labelling. The method is very sensitive, as concentrations in the picomolar range may be determined. The limit for the detection is around 200 Da, depending from the instrument used. Smaller molecules may also be detected with competition type assay or by including one or more amplification steps, which lowers the sensitivity of detection to femtomolar levels [17]. Finally, the technology can be included in portable instruments [18–20]. Field detection is desirable in monitoring and identification of biological agents as well as environmental pollutants. The portable SPR platforms were among other included in detection of ricin [21], enterotoxin B [22], 2,4-dichlorophenoxyacetic acid [23] and atrazine [24].

**Figure 1.**
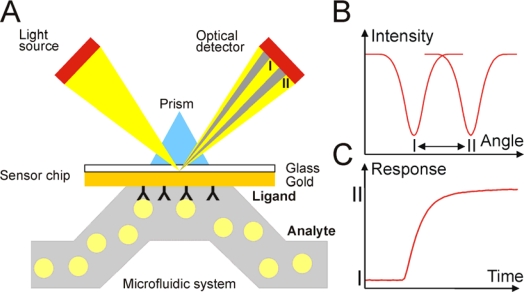
(A and B) The angle of the minimum of reflected intensity shifts when one molecule binds to the other one attached to the surface of the gold layer and, therefore, changes the refractive index of solution. In the SPR biosensor terminology the molecule attached to the sensor chip is termed ligand, and the other one analyte. (C) The sensorgram is a plot of the change of the SPR angle against time. It displays the real-time progress of the interaction at the sensor surface.

Biosensors based on SPR utilize a thin metal film between two transparent media of different refractive index, e.g. a glass prism and sample solution. Gold is preferably used in many SPR refractometers. A plane-polarized light beam entering the higher refractive index medium (glass prism) can undergo total internal refraction above a critical angle of incidence. Under these conditions, an electromagnetic field component of the light, the evanescent wave, will penetrate into the gold film. At a specific angle of incidence, interaction of this wave with free oscillating electrons at the gold film surface will cause the excitation of surface plasmons, resulting subsequently in a decrease in the reflected light intensity. This phenomenon is called surface plasmon resonance and occurs only at a specific angle of incident light. SPR system thus detects changes in the refractive index of the surface layer of a solution in contact with the sensor chip (Figure 1A).

**Figure 2.**
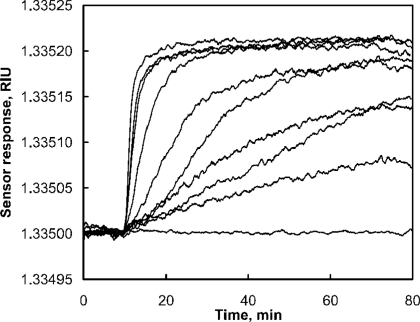
Direct detection of *Staphylococcus aureus* enterotoxin B. Polyclonal anti-SEB antibodies were immobilized on the sensing channel, while anti-dinitrophenol antibodies were immobilized on the reference channel. The reference channel was used to compensate for non-specific binding, bulk refractive index changes and temperature fluctuations. The concentration injected were 0, 0.2, 0.5, 1, 2, 3, 10, 25, 50 and 75 nM (from the bottom to the top). From Neimushin *et al.* [17].

SPR is observed as a sharp dip in reflected intensity at a certain angle, which is dependent on the refractive index of the medium on the non-illuminated side of the surface. This angle shifts when biomolecules bind to the surface and change the refractive index of the surface layer (Figure 1B). The sensorgram is a plot of the SPR angle against time, and displays the real-time progress of the interaction at the sensor surface (Figure 1C). Most of the SPR biosensors utilize response units (RU). The signal is proportional to the amount of the bound molecule (Figure 2). For proteins it was estimated that approximately 1,000 RU corresponds to surface coverage of 1 ng/mm^2^ [25].

The SPR signal is sometimes not large enough to detect analytes at the desired level with an SPR sensor, and this may be a problem when detecting small molecules. The localized surface plasmon resonance effect may be employed, by using Au nanoparticles as a further signal amplification approach [26]. For example, substrate-selective polymer gels with gold nanoparticles as the molecular recognition elements were designed. The sensing is based on swelling of the polymer gel that is triggered by an analyte binding. This increases the distance between the gold nanoparticles and substrate, which causes the shift of an SPR dip [27]. This approach have been recently used in atrazine detection. Detection of 5 pM atrazine in acetonitrile was achieved [28].

### 3.2. Instrumentation

The majority of publications using commercial SPR biosensor platforms employed Biacore technology [15,29], although other products also exist on the market that employ different optical and liquid-handling formats [30]. The essential components of a Biacore analytical system are the sensor chip, optical detector and an integrated microfluidic cartridge for the delivery of the sample to the surface of the sensor chip. Sensor chips are glass slides with a thin layer of functionalized gold. Biacore offers large variety of different sensor chips, that allow capture of almost any molecule. Hence, Biacore biosensors are readily used in basic science to study protein-protein, protein-DNA, protein-membrane, protein-small molecule, small molecule-membrane, etc., interactions [15,29,31]. The SPR imaging technology on array-based systems takes SPR analysis one step further. Biacore’s FLEXChip is an array-based system which can monitor up to 400 reactions simultaneously. In SPR imaging instruments, the SPR angles of many regions of interests may be monitored simultaneously by camera, which optically image the sensor surface.

### 3.3. Choice of Ligands

The successful detection of toxins by SPR requires a binding partner. Preferentially, this should be a molecule which recognizes the toxin with great specificity and high affinity. Non-specific binding and cross-reactivity of other toxins or contaminants in the sample may largely be prevented by the careful choice of a binding partner. Examples presented in the literature mostly use antibodies specific for a certain toxin (e.g. reference [32]). Other molecules from the cell that are bound by toxins may also be used. Nakamura *et al.* have used a photosynthetic reaction centre of *Rhodobacter sphaeroides* to specifically detect atrazine [33]. Fonfria *et al.* have employed phosphodiesterases to detect yessotoxin, since it was shown that yessotoxin activates them in cells [34]. And finally, glycolipids were used as ligands for specific detection of ricin, due to the fact that ricin binds sugars present on cell-surface [35].

It is possible that toxins may non-specifically bind to the components of the sensor chip, i.e. to the dextran matrix and molecules associated with it. However, this should be accounted for by optimization experiments, where different types of sensor chips, buffers, additives, etc. are tested. It is also well-known that ligand immobilization on the sensor chip may affect unspecific binding. This may be accounted for by employing a different immobilization strategy.

### 3.4. Assay Formats

**Figure 3.**
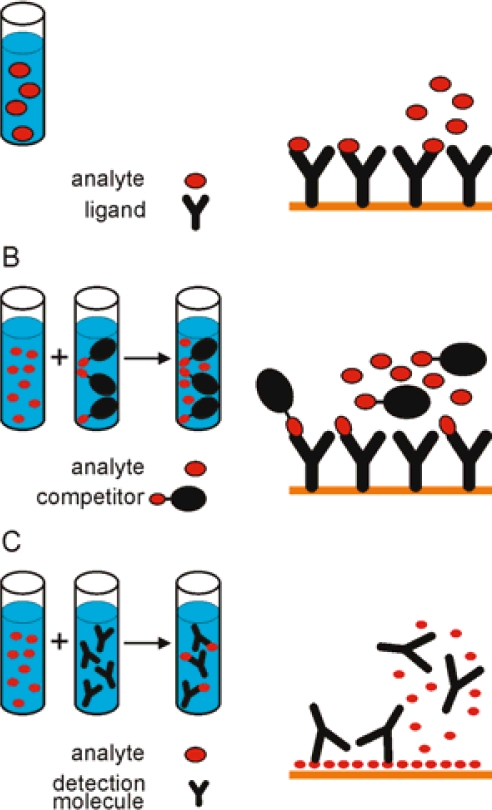
(A) Direct measurements are appropriate with molecules that give significant responses even at low molar concentrations. Two types of competition assays are surface competition method (B) or solution competition method (C), where the analyte is premixed with the competitor or detecting molecule, respectively.

In SPR the detector response is proportional to the mass of the analyte that binds to the ligand and, therefore, the responses resulting from binding of small molecules are low. SPR offers high sensitivity, the limit of detection for examples of toxin detection presented in this paper are in μM - nM concentration range. With the amplification it is possible to extend the limit of detection to pM - fM levels (see below). Direct measurement of the analyte may be performed when the analyte is large enough to produce significant responses even at low molar concentrations. Naimushin *et al.* showed direct detection of *Staphylococcus aureus* enterotoxin B (SEB) (Figure 2). The toxin with molecular weight 28.400 Da was detected at concentrations above 0.5 nM [17]. Competitive approaches may be more suitable for the analysis of small molecules (Figure 3). Two types of competitive assay formats are commonly used.

**Figure 4.**
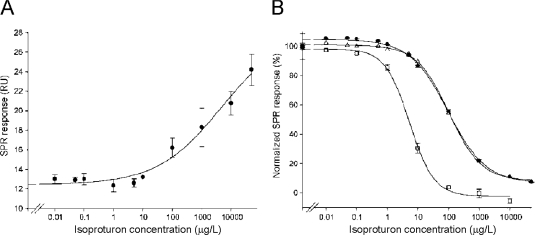
(A) The calibration curve of isoproturon generated in the direct detection SPR assay. (B) The calibration curves of isoproturon in the competitive assay for three different densities of the ligand on the surface of the chip. Adapted from Gouzy *et al.* [32].

Surface competition methods employ a larger analogue of an analyte, usually a protein conjugate, which is used as a competitor for binding to immobilized ligand on the sensor surface. The second approach is a solution competition method, where a known amount of a detecting molecule, e. g. an antibody, is mixed with the analyte, and the amount of free detecting molecule remaining in the mixture is measured. In both competition approaches, the response obtained is inversely related to the concentration of analyte in the sample. The maximum signal is obtained when no free analyte is present. The calibration curve is constructed from the responses of the known concentrations of the analyte (Figure 4).

Both approaches were used in isoproturon determination [32]. The direct binding of various herbicide concentrations to the surface, immobilized with anti-isoproturon monoclonal antibodies, is shown in Figure 4A. However, the SPR response due to the binding of isoproturon was measured only for concentrations above 10 μg/mL. The surface competition method was found to enhance the detection. Isoproturon-BSA conjugate was used as a competitor in the indirect detection approach. After optimizing the conjugate concentration, the calibration curves were obtained using three different ligand densities on sensor surface (Figure 4B). The lower ligand density was shown to be more sensitive to isoproturon [32].

## 4. Examples of Toxin Detection by SPR

SPR was successfully used for detection of bacterial and dinoflagellate toxins, mycotoxins and plant toxins. Application in control of chemicals of anthropogenic source in drinking water or food was also described. The use of pesticide and herbicide in agricultural industry has grown over last decades. These chemicals can remain for long periods in the environment and can contaminate the ground water, if not used correctly. The detection *in situ* is very convenient for the detection of these toxins and, therefore, portable biosensors based on SPR were developed recently.

### 4.1. Detection of Mycotoxins

Most of the toxins that are detected with SPR have low molecular weight and indirect tests offer better detection limits for their detection in samples. Several assays employing SPR for measuring mycotoxin concentration were described (Table 1). The indirect test was used for the determination of aflatoxin B1 [36]. Aflatoxins are highly toxic fungal secondary metabolites from *Aspergillus* species, which may contaminate foodstuffs and feeds. A conjugate consisting of aflatoxin B1 and bovine serum albumin was immobilized on the dextran gel surface. The samples mixed with known concentration of polyclonal antibody against aflatoxin B1 were injected over the surface. When the concentration of aflatoxin B1 in the samples was high, the response signals were low. Such inhibition assay enabled detection of four different mycotoxins simultaneously within a time frame of 25 minutes, including the time needed for sensor regeneration [36].

Daly *et al.* [37] produced a polyclonal anti-aflatoxin B1 antibody, which could be regenerated after the binding of the analyte by using an organic solution consisting of ethanolamine with acetonitrile, and was, therefore, usable for detection of several samples. An SPR immunosensor was developed to determine concentrations of the fumonisin B1 in spiked samples. After optimization of the antibody overlayer surface, a detection limit of 50 ng/mL was obtained for the direct assay with an analysis time under 10 min [38]. A rapid deoxynivalenol specific biosensor assay was developed based on a simple procedure that includes extraction with acetonitrile and 10-fold dilution with the running buffer. The majority of the assay results were in agreement with LC-MS/MS results [39].

### 4.2. Detection of Toxins and Toxic Substances of Anthropogenic Source

A broad range of synthetically produced chemicals can represent danger to health of humans or other organisms (Table 2). It includes herbicides, pesticides, antibiotic residues, etc. Atrazine is a widely used herbicide for growth control of grasses and crops. Farre *et al.* used a portable biosensor platform to detect atrazine in water with limit of detection at 20 ng/mL [24]. In another study, a direct detection system for atrazine inhibiting photosynthetic electron transfer was developed using the photosynthetic reaction centre from the purple bacterium. It was shown before that atrazine interacts with the D1 protein of the photosynthetic reaction centre [40]. Due to structural and functional homologies between plant and purple bacterial reaction centres the later was used in atrazine detection. Atrazine was detectable in the range from 1 to 100 mg/mL within 1 min. The obtained detection limit in this system, 1 mg/mL atrazine, was not enough to detect the guideline concentration in drinking water (2 ng/mL, World Health Organization). The authors suggest to improve the sensitivity with the competitive approach [33].

A simple SPR immunosensor, enabling parallel analysis of multiple analytes or multiple samples of an analyte, has been investigated for detection of a low-molecular-weight toxic substance, 2,4-dichlorophenoxyacetic acid. Low detection limit of 0.1 ppb (ng/mL) was established [23].

Gouzy *et al.* described the sensor for monitoring isoproturon, a selective and systemic herbicide. The detecting rat monoclonal anti-isoproturon antibody was reversibly immobilized through the use of a capture mouse anti-rat monoclonal antibody, which was covalently immobilized on the sensor chip surface. The capture of the anti-isoproturon antibody could be carried out up to 120 times (immobilization/regeneration cycles) without any evidence of activity loss. Using a strategy based on a surface competition assay easily enhanced the limit of detection. The sensitivity and working range of the indirect format were found to be dependent on the surface density of the anti-isoproturon monoclonal antibody. As expected for competitive formats, the lowest surface coverage allowed a lower detection of the herbicide isoproturon with a calculated limit of detection of 0.1 μg/L [32] (Figure 4).

A rapid and sensitive biosensor immunoassay was developed for residues of the antiparasitic agent ivermectin in bovine liver. A detection limit of 19.1 ng/g was achieved [41].

The antibiotics streptomycin and dihydrostreptomycin are used in modern agriculture for the treatment of bacterial infections in cattle, sheep, pigs and poultry. Their due detection in food is important because antibiotic residues could be harmful to consumers. The assay allowed the direct analysis of bovine whole milk, honey samples required dilution with buffer, while kidney and muscle samples from pigs were homogenized in an aqueous extraction buffer and clarified by centrifugation [42].

In recent years there has been an increase in the use of tylosin in apiculture due to resistance to oxytetracycline. Mass spectrometry based methods have been developed for the detection, but up until now there has been no complementary screening method available, capable of sub 10 μg/kg detection limits. Caldow *et al.* reported the assay with the detection limit 2.5 μg/kg [43]. Honey samples containing trace residue levels of tylosin were analyzed by both the biosensor screening method and an LC-MS/MS confirmatory procedure. The results were in good agreement [43].

The wide range of kits for screening for some other drug residues is commercially available on the market. They offer rapid, precise and highly sensitive determination. Studies have demonstrated that the results obtained with these kits correlates well with other confirmatory methods (http://www.biacore.com/food/products/biacore_q/qflex_kits/index.html).

An interesting approach for the detection of phenol, employing living cells, was presented by Choi *et al.* [44]. They fabricated the sensor surface with *E. coli* O157:H7 strain and exposed such surface to phenol. The damage following the injection of phenol had a significant impact on the total amount of intracellular biomaterials in attached cells, which induced the change of SPR signal. The detection limit was determined to be 5 ppm of phenol [44].

### 4.3. Detection of Dinoflagellate Toxins

The mouse bioassay is most widely used to detect biotoxins produced by marine dinoflagellates in shellfish samples. Alternative methods of toxin detection were developed, due to animal welfare concerns. Fonfria *et al.* developed a new detection and quantification method for yessotoxin. The assay is based on the interaction of toxin with phosphodiesterase enzyme, which was immobilised on the sensor surface [34]. Detection protocols for okadaic acid [45] and saxitoxin [46] were also established. When the concentrations of okadaic acid determined with the biosensor method were compared with the values obtained by LC–MS in contaminated shellfish samples, the correlation between the two analytical methods was found to be highly satisfactory (r^2^=0.991) [45].

### 4.4. Detection of Bacterial Toxins

Bacterial and plant toxins have large molecular weight and can be detected directly when using SPR. Enterotoxin B is an exotoxin produced by *Staphylococcus aureus*. It is one of the toxins responsible for staphylococcal food poisoning in humans and has also been produced as a biological weapon. The toxin was determined in different samples, including milk, seawater and mushrooms (Table 4). Naimushin *et al.* designed the SPR platform that is ideal for the field use. The enterotoxin was detectable at sub-nanomolar levels and with amplification at femtomolar levels [17].

The SPR immunoassay was compared with an ELISA and the traditional bioassay for *Clostridium perfringens* β-toxin. The SPR detection proved to be less sensitive than ELISA, however, the sample treatment lasted only 20 min, while with ELISA it lasted six hours [47]. Tetanus toxin is large protein with molecular weight 150 kDa. Due to its large size its direct detection is easy even in samples with low concentrations [48].

### 4.5. Detection of Plant Toxins

Ricin represents one of the most potent plant toxins. Its detection has been performed by many methods, but not with SPR until recently. Feltis *et al.* developed a hand-held biosensor. They were able to detect ricin at 200 ng/mL, which is approximately 2,500 times less than the minimum lethal dose, in 10 min [21].

Two additional SPR-based assays for ricin detection were published in 2008. Tran *et al.* identified two suitable antibodies with strong affinity to all six ricin variants. The assay was linear over a wide range of ricin concentrations (up to at least 750 ng/mL) with a limit of detection of 0.5 ng/mL [49]. The fact that ricin binds cell-surface oligosaccharides was employed in another assay. Analysis using carbohydrate probes allowed researchers to detect the toxin in a highly sensitive and facile manner (10 pg/mL, 5 min) [35].

Chinowsky *et al.* have developed multi-analyte SPR instrument and showed detection of ricin A among other five distinct analytes ranging from small molecules to whole microbes during the course of a single experiment [22].

Abrin is a highly potent and lethal type II ribosome inactivating toxin from *Abrus precatorius*. It is very similar to ricin in structure and biochemical mechanism of action, but has been stated to be approximately 75 times more toxic in mice. The authors described the selection and characterization of two human monoclonal antibodies, which are capable of binding native abrin with high affinity and specificity. The limit of detection was 35 and 75 ng/mL [50].

## 5. Conclusions

SPR assays have been developed for detecting and identification of various toxins. Detection is possible in water and food samples with detection limits as low as femtomolar range. Some advantages of SPR assay, such as no need for molecular labeling, low sample consumption and speed of detection, makes this technology suitable for fast screening. Future research will probably aim more at improving sensor performance of portable platforms, which enable rapid determination of possible toxic molecules on-site.

## Figures and Tables

**Table 1.** Mycotoxin detection.*

**Toxin**	**M_W_ (Da)**	**Type of detection**	**Detection limit**	**Reference**
Deoxynivalenol	296.3	indirect	0.5 ng/g	[36]
Deoxynivalenol	296.3	indirect	2.5 ng/mL	[39]
Aflatoxin B1	312.3	indirect	0.2 ng/g	[36]
Aflatoxin B1	312.3	indirect	3 ng/mL	[37]
Zearalenone	318.4	indirect	0.01 ng/g	[36]
Ochratoxin A	403.8	indirect	0.1 ng/g	[36]
Ochratoxin A	403.8	direct	0.1 μg/mL	[51]
Fumonisin B1	721.8	indirect	50 ng/g	[36]
Fumonisin B1	721.8	direct	50 ng/mL	[38]

* The data presented in this and subsequent tables are listed as specified in indicated references

**Table 2.** Detection of toxic molecules from water and food, mostly of anthropogenic source.

**Toxin / toxic molecule**	**M_W_ (Da)**	**Type of detection**	**Detection limit**	**Reference**
Phenol	94.1	direct	5 ppm	[44]
Simazine	201.7	indirect	0.11 μg/L	[52]
Carbaryl	201.2	indirect	1.38 μg/L	[53]
Isoproturon	206.3	indirect	0.1 μg/L	[32]
Atrazine	215.7	indirect	20 ng/L	[23]
Atrazine	215.7	direct	1 μg/mL	[33]
2, 4-Dichlorophenoxyacetic acid	221	indirect	0.1 ng/mL	[23]
2, 4-Dichlorophenoxyacetic acid	221	indirect	0.5 ng/mL	[54]
Benzo[a]pyrene	252.3	indirect	0.01 ppb	[55]
Sulfamethazine	279.3	indirect	1 ppb	[56]
Chlorpyrifos	350.6	indirect	0.05 μg/L	[53]
DDT	354.5	indirect	15 ng/L	[53]
Streptomycin	581.6	indirect	15 μg/kg	[42]
Dihydrostreptomycin	583.6	indirect	n.d. *	[42]
Ivermectin	875.1	indirect	19.1 ng/mL	[41]

* n.d., not determined

**Table 3.** Detection of Dinofllagelate toxins.

**Toxin**	**M_W_ (Da)**	**Type of detection**	**Detection limit**	**Reference**
Saxitoxin	299.3	indirect	2 ng/mL	[46]
Domoic acid	310	indirect	0.1 ng/mL	[57]
Okadaic acid	805	indirect	126 ng/g	[45]
Yessotoxin	1,134	direct	1mg/kg	[34]

**Table 4.** Bacterial toxin detection.

**Toxin**	**M_W_ (Da)**	**Type of detection**	**Detection limit**	**Reference**
Enterotoxin B	28,400	direct	1.96 ng/mL	[17]
			0.14 ng/mL (one amplification step)	
			0.0028 ng/mL (two amplification steps)	
Enterotoxin B	28,400	direct	n.d. *	[22]
Enterotoxin B	28,400	direct	10 ng/mL	[58]
Enterotoxin B	28,400	direct	10 ng/mL	[59]
β-toxin	35,000	direct	n.d.	[47]
Tetanus toxin	150,000	direct	0.028 Lf/mL	[48]

* n.d., not determined

**Table 5.** Plant toxin detection.

**Toxin**	**MW (Da)**	**Type of detection**	**Detection limit**	**Reference**
Ricin A	32,000	direct	n.d. *	[22]
Ricin	60,000	direct	200 ng/mL	[21]
Ricin	60,000	direct	0.5 ng/mL	[49]
Ricin	60,000	direct	1.7 μg/mL	[35]
Abrin	65,000	direct	35; 75 ng/mL	[50]

* n.d., not determined
